# Sufficiently activated mature natural killer cells derived from peripheral blood mononuclear cells substantially enhance antitumor activity

**DOI:** 10.1002/iid3.1143

**Published:** 2024-01-10

**Authors:** Chuanling Liu, Yingying Li, Yanrong Li, Meng Hu, Haiyan Wang, Shasha Lu, Zhao Li, Dilinuer Dilimulati, Shunchang Jiao, Shelian Lu, Weihong Zhao

**Affiliations:** ^1^ Department of Oncology Chinese PLA General Hospital Beijing China; ^2^ Research and Development Department Beijing DCTY® Biotech Co., Ltd Beijing China

**Keywords:** ascites, natural killer cell, ovarian cancer, peripheral blood

## Abstract

**Background:**

Peripheral blood‐derived natural killer (NK) cells spontaneously lyse tumor cells without prior sensitization. However, NK cells in peripheral blood (PBNK cells) are in a resting state and exhibit inhibitory phenotypes and impaired cytotoxicity. Thus, strengthening the cytotoxic effector function of PBNK cells and improving NK cell expansion in vitro for a convenient allogeneic therapy are essential.

**Materials and Methods:**

Pure cytokine activation and expansion of NK cells (super NK [SNK]) from peripheral blood mononuclear cells were studied. Markers of activated and inhibited NK cells and cytokine secretion by NK cells were examined using flow cytometry. NK cell antitumor activity in vitro was assessed using lactate dehydrogenase (LDH) cytotoxicity assay and an Incucyte real‐time imaging system. Additionally, the function of SNK cells against ascites caused by ovarian cancer in NOD‐Prkdc(em26Cd52)il2rg(em26Cd22)/Nju (NCG) mice was determined. In a further investigation of the differences between PBNK and SNK, the mRNA of both cells was sequenced and analyzed.

**Results:**

Human peripheral blood mononuclear cells showed selective NK cell expansion upon cytokine activation and culture. Both SNK and PBNK cells expressed activation markers, but at different levels, and SNK cells secreted more cytokines related to cytotoxicity than PBNK cells did. Accordingly, SNK cells exhibited strong antitumor activity ex vivo and improved NCG mice survival after intraperitoneal ovarian cancer transplantation. Mechanistically, SNK cells expressed more genes associated with nucleotide metabolism, fatty acid, and ATP metabolism than PBNK cells.

**Conclusion:**

SNK cells derived from peripheral blood mononuclear cells have sufficiently activated mature characteristics and high antitumor activity, rendering them a highly promising and essential therapeutic approach for cancer treatment.

## Graphical abstract

Super natural killer cells derived from peripheral blood mononuclear cells have sufficiently activated mature characteristics and high antitumor activity, rendering them a highly promising and essential therapeutic approach for cancer treatment.

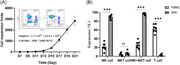

## 1 INTRODUCTION

Human natural killer (NK) cells belong to the innate lymphoid cell family and are derived from CD34^+^ precursors that originate from hematopoietic stem cells.^1^, ^2^ They are distributed in the peripheral blood, bone marrow, and tissues and are identified by the absence of surface T cell receptors and associated CD3 molecules and by the expression of the neural cell adhesion molecule (also known as CD56).^3^ Unlike T cells, NK cells can directly recognize and rapidly kill virus‐infected cells and tumor cells without antigen pre‐sensitization and are not restricted by major histocompatibility complexes, which do not cause graft‐versus‐host disease^4^, ^5^, ^6^, ^7^; thus, they are ideal candidates for adoptive immunotherapy.^8^ NK cells are promising as “off‐the‐shelf” cell therapy products for anticancer drugs.

NK cells have a unique mechanism to discriminate between cancer and healthy cells; this mechanism relies on a balance between stimulatory and inhibitory receptors.^2^, ^9^, ^10^ Studies have observed that when a set of inhibitory killer cell immunoglobulin‐like receptors bound with the major histocompatibility complex class I molecules of healthy cells, the function of NK cells was suppressed, and the destruction of fine self‐tissue was minimized.^8^ Other studies have observed that natural cytotoxicity receptors (e.g., NKp30, NKp44, and NKp46), NK‐activating receptors (e.g., sDNAM‐1 and NKG2D), and activating killer cell immunoglobulin‐like receptors were bound by the overexpression ligands of cancer cells and that NK cells were activated and produced many cytotoxic granules and pro‐inflammatory factors for lysing cancer cells.^11^ Moreover, activated NK cells release cytolytic granules, including the pore‐forming protein perforin, granzymes,^12^, ^13^ and granulysin,^14^, ^15^ to kill target cells and secrete cytokines and chemokines, shaping innate and adaptive immune responses.^16^ Finally, upregulation of the Fas ligand (FasL) in NK cells induces apoptosis in Fas‐expressing tumor cells through the Fas‐FasL pathway, a key death factor in NK cell antitumor activity.^17^, ^18^

Malignant ascites (MA) is the most commonly observed complication of advanced ovarian cancer, cirrhosis, and acute pancreatitis. MA has a poor prognosis; significantly reduces quality of life; and increases abdominal distention, pain, and dyspnea.^19^, ^20^, ^21^, ^22^ In addition, ascites induces metastasis and chemoresistance and decreases tumor resectability.^23^, ^24^ In recent years, immunotherapy has been used as an innovative treatment for MA but has not been as successful as expected owing to the vast heterogeneity of MA.^25^ Notably, NK cells have the unique characteristic of recognizing pan‐specific tumor cells and killing tumor cells through direct or indirect pathways,^26^, ^27^ implying that NK cells may be the best choice for the treatment of MA. Thus, in this study, we comprehensively evaluated the characteristics of the ex vivo expansion of NK cells derived from peripheral blood mononuclear cells (PBMCs) and their anti‐MA activity in ovarian cancer.

## 2 MATERIALS AND METHODS

### 2.1 Cell lines and animals

Human non‐small cell lung cancer cell lines H358 and A549 and human ovarian cancer cell lines SKOV‐3 and OVCAR‐8 were purchased from the American Type Culture Collection. The H358, A549, and SKOV‐3 cells were lentivirally transduced to express green fluorescent protein, and the OVCAR‐8 cells were transduced to express luciferase.

Female NOD‐Prkdc(em26Cd52)il2rg(em26Cd22)/Nju (NCG) mice were supplied by GemPharmatech Co., Ltd. The NCG mice were used for the in vivo experiments and randomly assigned to experimental groups after tumor cell inoculation. The mice were housed in accordance with the requirements for specific pathogen‐free‐grade animals (SYXK [Beijing] 2019‐0028) in standard cages (*n* = 6 per cage) at 23 ± 3°C on a 12/12 h light–dark cycle in a clean room with humidity levels ranging from 40% to 70%. Food and water were supplied ad libitum. All mouse experiments were conducted according to Institutional Animal Care and Use Committee‐approved protocols.

### 2.2 Ex vivo NK cell expansion protocol

Sufficiently activated mature NK cells (super NK [SNK]) were expanded from healthy donor PBMC using a DCTY NK serum‐free medium culture kit (LY1201; Beijing DCTY Biotech Co., Ltd). During the cell culture, the medium was supplemented with 8% CTS‐immune cell SR (Gibco). In brief, the culture flask was coated with NK reagent A at 4°C for 24 h. The coating fluid was discarded, and the PBMCs were inoculated with a medium containing reagent B. In the first week of cell culture, the medium was added to the cells with NK reagent C. Subsequently, the cells were cultured in a medium containing NK reagent D. The ex vivo SNK cells were continuously cultured until Days 18–21, and the SNK cells with a purity of exceeding 90% were used for further in vitro experiments.

### 2.3 Animal treatment and grouping

For the intraperitoneal ovarian cancer ascites models, 1 × 10^7^ OVCAR‐8 tumor cells were injected into the abdomen of 6‐ to 8‐week‐old female immunodeficient NCG mice.

In the dose‐escalation study, a total of 30 mice were randomly divided into control group, 1 × 10^7^ cells group, 2 × 10^7^ cells group, 4 × 10^7^ cells group, and 8 × 10^7^ cells group, with *n* = 6 per group. In multiple doses trials, 12 mice were randomly divided into two groups: the control group (*n* = 6) and the SNK group (*n* = 6). Random numbers were generated using the standard = RAND() function in Microsoft Excel.^28^ Different doses of SNK cells were transplanted into the mice via intraperitoneal injection. Multiple doses were administered once per week. An equivalent volume of saline was injected into the abdomen, which served as a control. Tumor burden in the mice was monitored using a bioluminescence imaging system (IVIS Lumina Series III, PerkinElmer) weekly after SNK injection. The abdominal circumference, body weight, and survival indicating ascites volume and mouse living state, were also monitored weekly. Furthermore, the animals were observed daily to identify any potential complications or adverse reactions subsequent to the treatment. The survival of the animals was documented based on the criteria established for humane endpoints. Humane endpoints included loss of motor function, moribund state, inability to drink/feed, and paralysis. No data or animals were excluded from the analysis. All the operations or measurements of mice were performed in the dedicated laboratory space.

For each animal, four different investigators were involved as follows: a first investigator administered the treatment based on the randomization rule. This investigator was the only person who knew the group allocation. A second investigator was responsible for giving cells or solutions for the mice, whereas a third investigator performed the measurement procedure. Finally, a fourth investigator analyzed the data of results. This study protocol was not registered.

### 2.4 NK cell isolation from PBMC

NK cells in peripheral blood (PBNK cells) were isolated from peripheral blood using a Rosette SepTM Human NK Cell Enrichment Cocktail kit (STEMCELL Technologies) according to the manufacturer's instructions.

### 2.5 Flow cytometry

For detecting NK cell‐activated and inhibitory surface marker expression, cells were stained with conjugated anti‐human antibodies as described previously.^29^ Samples were analyzed using a BD LSRII flow cytometer (BD Biosciences) and FlowJo software (Tree Star). CD56^+^CD3^−^ populations were evaluated in the NK cells. NK cell markers were determined using the following antibodies: FITC anti‐human CD3 (FITC, 981002, BioLegend), APC anti‐human CD56 (APC, 304610, BioLegend), PE anti‐human CD69 (KIRD1) (PE, 981002, BioLegend), PE anti‐human CD336 (NKP44) (PE, 325108, BioLegend), PE anti‐human CD159c (NKG2C) (PE, 375004, BioLegend), PE anti‐human CD314 (NKG2D) (PE, 320805, BioLegend), PE anti‐human CD94 (PE, 305506, BioLegend), PE anti‐human CD335 (PE, NKp46, BioLegend), PE anti‐human CD96 (TACTILE) (PE, 338406, BioLegend), and PE anti‐human CD366 (TIM‐3) (PE, 345006, BioLegend).

### 2.6 Cytotoxicity assays

Cytotoxicity mediated by PBNK or SNK cells for 4 h was tested using the lactate dehydrogenase (LDH) assay, according to a previously reported protocol.^30^ Dynamic cytotoxicity was observed for 48 h using an Incucyte ZOOM.^31^ In brief, the tumor cells were seeded in a 96‐well plate at a density of 2.0 × 10^3^ cells/well in triplicate for 6 h, followed by the addition of PBNK or SNK cells (Effector‐to‐Target, E:T = 5:1). Images were acquired hourly. The cytotoxicity of the target cells was analyzed by measuring the number of green cells.

### 2.7 Secretory molecules assay

The determination of secretory molecules, including granzyme A (GZMA), granzyme B (GZMB), perforin, granulysin, secretory FasL (sFasL), interferon‐gamma (IFN‐γ), tumor necrosis factor‐alpha (TNF‐α), and interleukin (IL)‐17a secreted by the PBNK and SNK cells, were detected using the LEGENDplexTM Human CD8/NK Panel (Biolegend). In brief, the SNK or PBNK cells were washed two times with phosphate‐buffered saline and resuspended in basal medium at 1 × 10^6^ cells/mL. After 24 h, the supernatant was collected and examined according to the manufacture's protocol, and the data were analyzed using LEGENDplexTM8.0.

### 2.8 RNA isolation and sequencing

Total RNA from the PBNK and SNK cells was extracted using the TRIzol Reagent (Invitrogen) according to the manufacturer's protocol. RNA quality control, library preparation, sequencing, and data analysis were performed by Majorbio Bio‐Pharm Biotechnology Co., Ltd. RNA degradation and contamination were detected through 1% agarose gel electrophoresis. RNA was defined and qualified using an ND‐2000 (NanoDrop Technologies). Only high‐quality RNA samples were used to construct the sequencing libraries. The procedure was performed as described in the literature.^32^

### 2.9 Statistical analyses

GraphPad Prism version 9.0 (GraphPad Software) was used for statistical analysis. Two‐group comparisons were analyzed using a two‐tailed unpaired Student's *t* test, and multiple‐group comparisons were analyzed using one‐way ANOVA. Mouse survival curves were assessed using the log‐rank Mantel–Cox test. Differentially expressed genes with |log2FC| ≥ 1 and FDR ≤ 0.05 (DESeq. 2) or FDR ≤ 0.001 (DEGseq) were considered to be significantly different. In addition, Fisher's exact test and FDR correction for multiple testing were used to analyze the GO, KEGG, and REACTOM enrichments. Data are shown as mean ± SEM, and differences were considered significant if *p* < .05 (*p < .05; **p < .01; ***p < .001, ns = not significant).

## 3 RESULTS

### 3.1 SNK cells derived from human PBMCs showed preferable expansion

**Figure 1:**
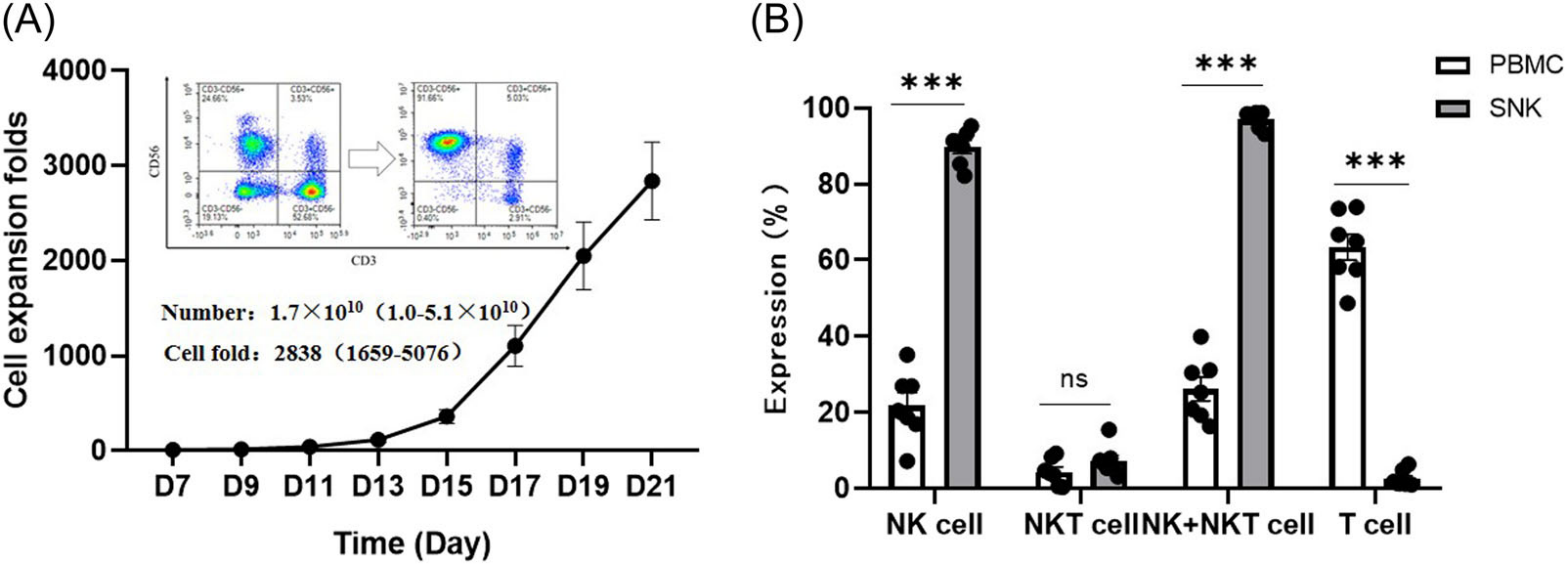
SNK cells derived from human PBMCs show preferable expansion. PBMCs were isolated from healthy donors and cultured using a pure cytokine stimulation NK expansion system. After culture for 18–21 days, cells were harvested and identified by flow cytometry. (A) A curve of the total cell expansion folds was generated during the culture. Data are shown as mean ± SE (*n* = 7). Inset images show the purity of NK cells before (Day 0) and after (Day 21) expansion from one representative experiment. (B) The proportion of different types of immune cells before and after PBMC expansion were determined for comparison using a two‐tailed paired Student's *t* test (mean ± SEM, *n* = 7). *p < .05, **p < .01, ***p < .001. NK, natural killer; ns, no significance; PBMC, peripheral blood mononuclear cell; SNK, super natural killer.

To obtain the required quantities of NK cells for infusions, we performed ex vivo expansion of NK cells. This research endeavors to expand SNK cells derived from PBMCs using a DCTY NK serum‐free medium culture kit. This kit utilizes a pure cytokine system without feeder cell coculture, which has been used previously and has proven effective in obtaining significant quantities of highly purified expanded CD3^−^/CD56^+^ NK cells derived from peripheral blood. After 18–21 days of culture in this system, the cells were harvested with thousands‐fold amplification (mean: 2838‐fold; range: 1659‐ to 5076‐fold) (Figure 1A), and flow cytometry was used to assess the purity of expanded SNK cells. The proportion of CD3^−^CD56^+^NK increased significantly, in which the ratio of CD56^+^ cells (NK + NKT) reached 97.08 ± 2.00% (range: 93.21%–98.78%). Additionally, the proportion of CD3^+^CD56^−^ T cells decreased substantially, from 63.40 ± 8.48% (range: 48.77%–73.97%) to 2.57 ± 1.97% (range: 1.03%–6.34%) (Figure 1B). These results demonstrate that an abundance of NK cells can be cultured from PBMCs using a pure cytokine system.

### 3.2 SNK cells demonstrated sufficient activation compared with PBNK cells

**Figure 2:**
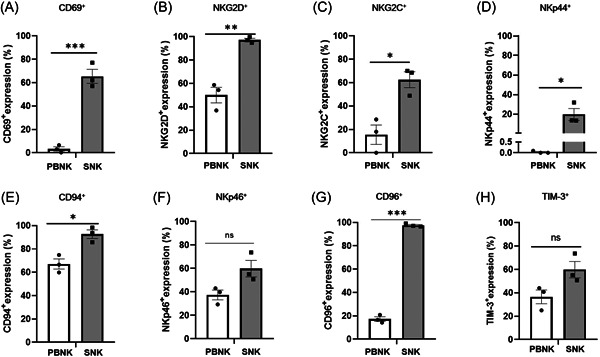
SNK cells show highly activated markers compared with PBNK cells. Flow cytometry was used to assess the surface expression of (A)–(F) activation and (G and H) inhibitory markers on the CD56^+^CD3^−^(NK cell) population derived from resting PBNK and expanded SNK cells on Day 21. Data (mean ± SEM) from three healthy donors. A two‐tailed unpaired Student's *t* test was used to compare surface resting and expanded NK cells from the same source. *p < .05, **p < .01, ***p < .001. NK, natural killer; ns, no significance; PBNK, NK in peripheral blood; SNK, super natural killer.

The presence of NK receptors impacts the function of NK cells, and to ascertain the activation status of SNK cells, we utilized flow cytometry to analyze the expression of both activating and inhibitory receptors on NK cells. The results demonstrated that, compared with the PBNK cells, the expression of activating receptor CD69 of SNK cells increased significantly, from 3.29 ± 2.58% to 65.40 ± 8.53% (Figure 2A). Similarly, the expression of NKG2D increased from 50.00 ± 9.43% to 97.17 ± 2.00% (Figure 2B). The other activating receptors, NKG2C, NKp44, CD94, and NKp46, were also upregulated after ex vivo expansion (Figure 2C–E), suggesting significant activation of SNK cells. However, the expression of inhibitory receptor CD96 was also significantly upregulated in SNK cells (Figure 2G), with the same tendency of TIM‐3 expression (Figure 2H).

### 3.3 SNK cells exhibited abundant molecule secretions compared with PBNK cells

**Figure 3:**
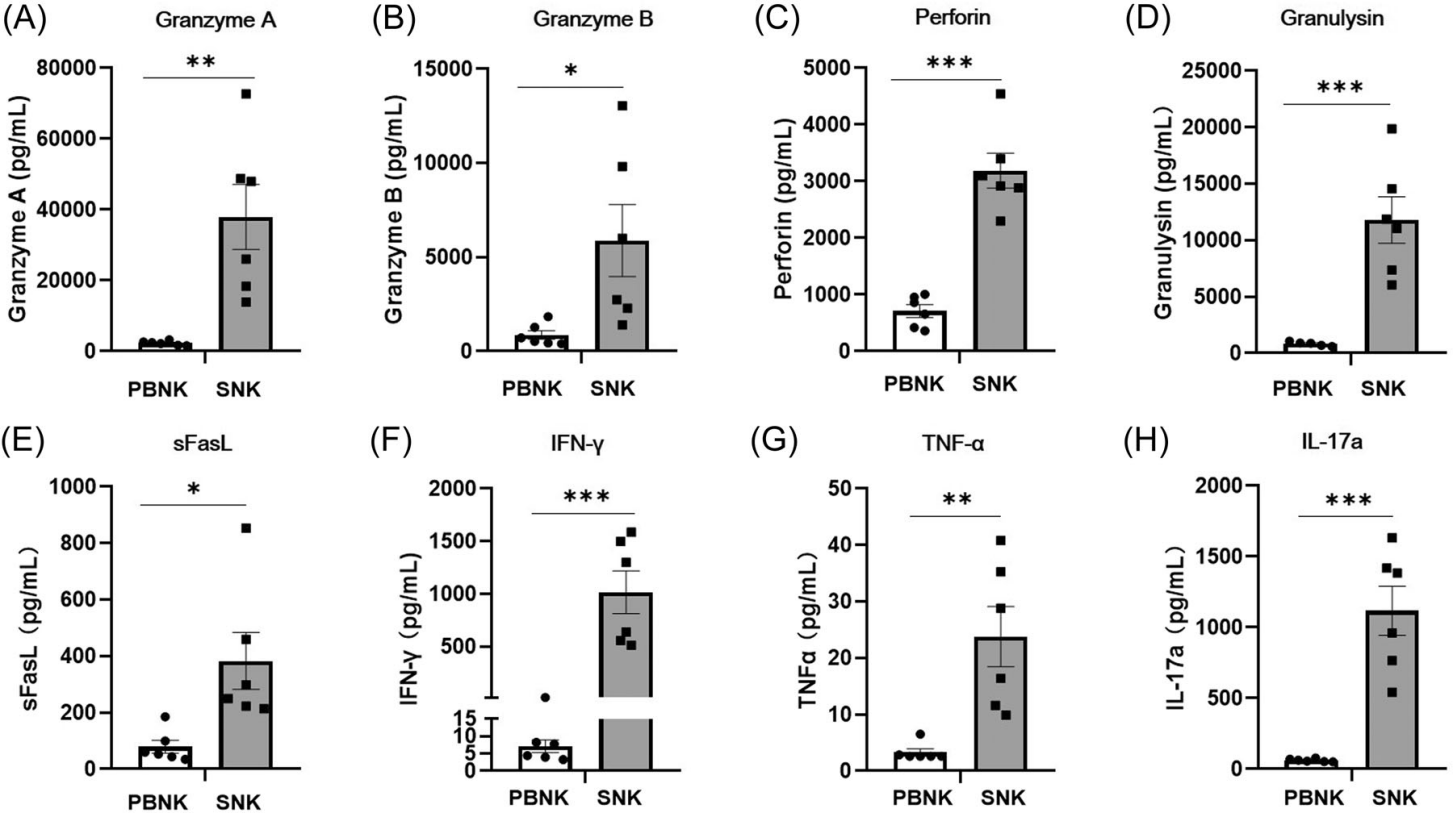
Abundant cytokine secretion by SNK cells for 24 h. Flow cytometry was conducted with the LEGENDplex™ Human Inflammation Panel (13‐plex) to evaluate the cytokines of resting PBNK cells (isolated from healthy human peripheral blood) and expanded SNK cells on Day 21. PBNK and SNK cells were stimulated for 24 h with cytokine‐free medium, and the supernatants were subsequently assayed for levels of (A) granzyme A, (B) granzyme B, (C) perforin, (D) granulysin, (E) sFasL, (F) IFN‐γ, (G) TNF‐α, and (H) IL‐17a. Statistical analysis was performed using one‐way ANOVA. Data are shown as mean ± SEM (n = 6). *p < .05, **p < .01, ***p < .001. NK, natural killer; ns, no significance; PBNK, NK in peripheral blood; SNK, super natural killer.

Secretory molecules of SNK and PBNK cells were analyzed through flow cytometry using the LEGENDplex Human CD8/NK panel. Notably, a significantly higher expression of direct cytotoxic effector‐related molecules, including Granzyme A (37532 ng/mL vs. 2295 ng/mL, *p* = .0013); Granzyme B (6238 ng/mL vs. 871 ng/mL, *p* = .0145); Perforin (3120 ng/mL vs. 711 ng/mL, *p* < 0.0001); Granulysin (10914 ng/mL vs. 1237 ng/mL, *p* = .0004); indirect cytotoxic effector‐related molecules including sFasL (414 ng/mL vs. 79 ng/mL, *p* = .0067); IFN‐γ (1182 ng/mL vs. 7 ng/mL, *p* = .0168); TNF‐α (45 ng/mL vs. 3 ng/mL, *p* = .1708), and immunoregulatory factor IL‐17a (1114 ng/mL vs. 61 ng/mL, *p* < 0.0001) were observed in SNK cells compared to that in PBNK cells (Figure 3); this finding implies that SNK cells have enhanced antitumor cytotoxicity after ex vivo expansion.

Additionally, the same assay was performed to investigate the soluble factors secreted by SNK and PBNK cells in the presence of target cancer cells H358, A549, and SKOV‐3 (Figures S1 and S2). Interestingly, when SKOV‐3 cells were cocultured with SNK cells, there was a notable elevation in the levels of the cytotoxic factors granzyme B and granulysin (Figure 2F,H). Comparable patterns were also observed for sFasL, IFN‐γ, and Perforin (Figure 2C,D,G).

### 3.4 SNK cells exhibited enhanced cytotoxicity in vitro

**Figure 4:**
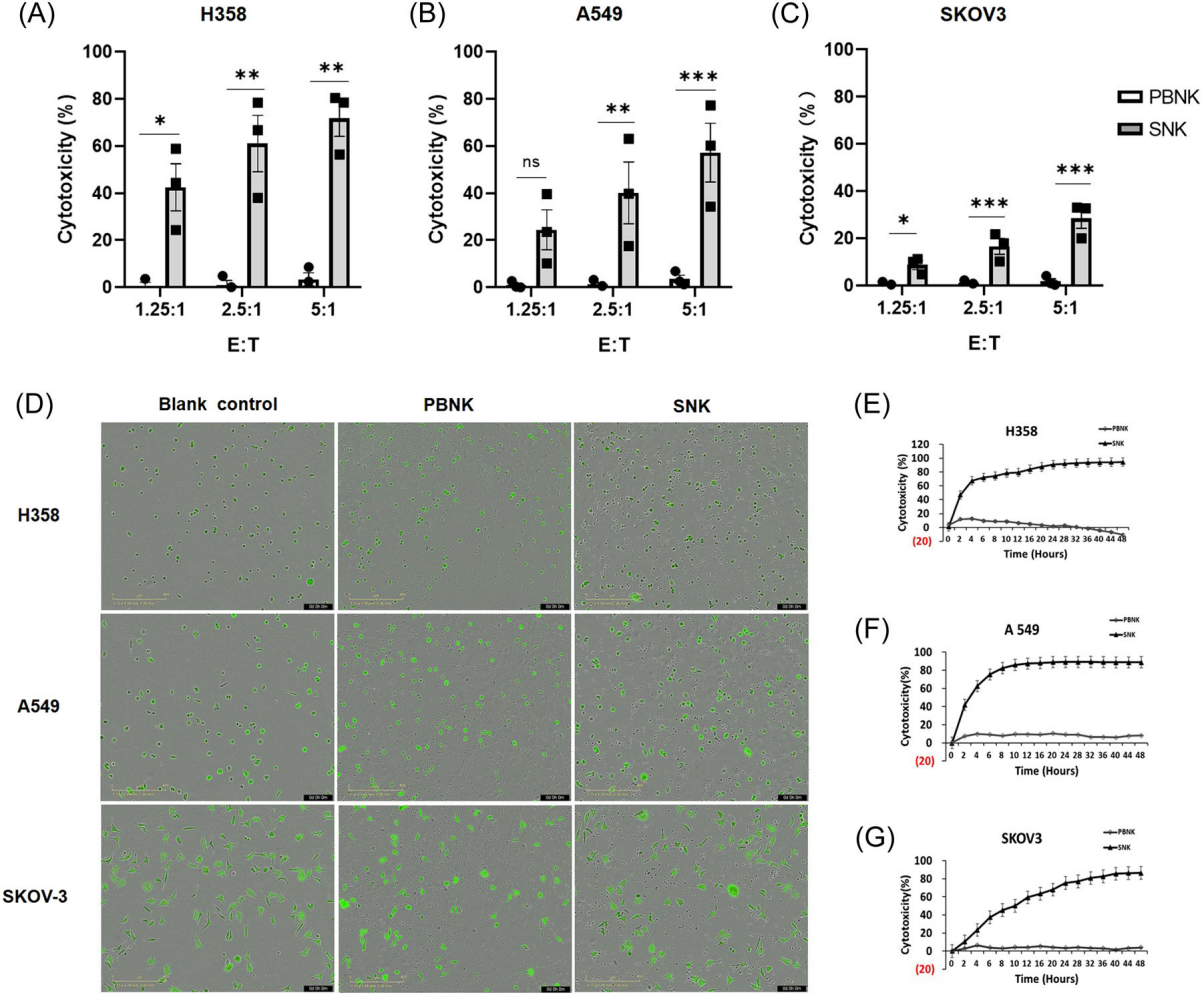
SNK cells have better cytotoxicity than PBNK cells against multiple cancer cell lines ex vivo. The Killing activity of resting PBNK cells (isolated from healthy human peripheral blood) and expanded SNK cells on Day 21 against (A) H358, (B) A549, and (C) SKOV‐3 cancer cell lines for 4 h were measured by LDH method (*n* = 3). (D) Dynamic imaging of cytotoxicity by SNK cells and PBNK cells against H358, A549, and SKOV‐3 for 48 h using the Incucyte real‐time imaging system at effector‐to‐target (E:T) = 5:1. The dynamic cytotoxicity analysis was performed, including (E) H358, (F) A549, and (G) SKOV‐3. Results are from three independent experiments. Data were analyzed using the two‐tailed unpaired Student's t test. Data are shown as mean ± SEM. *p < .05, **p < .01, ***p < .001. ns, no significance; PBNK, natural killer in peripheral blood; SNK, super natural killer.

The cytolytic function of SNK and PBNK cells against H358 and A549 (two non‐small cell lung cancer lines) and SKOV‐3 (ovarian cancer line) in vitro was evaluated for 4 h by LDH assay or 48 h dynamic cytotoxicity using an Incucyte ZOOM. The results indicated that the SNK cells exhibited a significant increase in cytotoxicity against H358 cells compared to PBNK cells in all three groups: group (E:T = 1.25:1) 42.52% versus 0.74%, *p* = .0145; group (E:T = 2.5:1) 61.1% versus 0.78%, *p* = .0078; and group (E:T = 5:1) 71.81% versus 3.38%, *p* < .0011. Furthermore, the SNK cells demonstrated a similar cytotoxicity effect on A549 lung cancer cells and SKOV‐3 ovarian cancer cells (Figure 4B,C). Subsequently, we utilized the Incucyte real‐time imaging system to dynamically visualize the cytotoxicity effect exerted by SNK cells and PBNK cells against H358, A549, and SKOV‐3 cell lines over 48 h while maintaining a ratio of E:T = 5:1 (Figure 4D–G). Notably, the activated SNK cells exhibited significantly higher cytotoxicity against tumor targets in the short‐ and long‐term killing activity assays than the PBNK cells (Figure 4A–G), indicating that SNK cells were sensitive to different types of tumor cell killing.

### 3.5 SNK cells displayed improved antitumor activity in vivo

**Figure 5:**
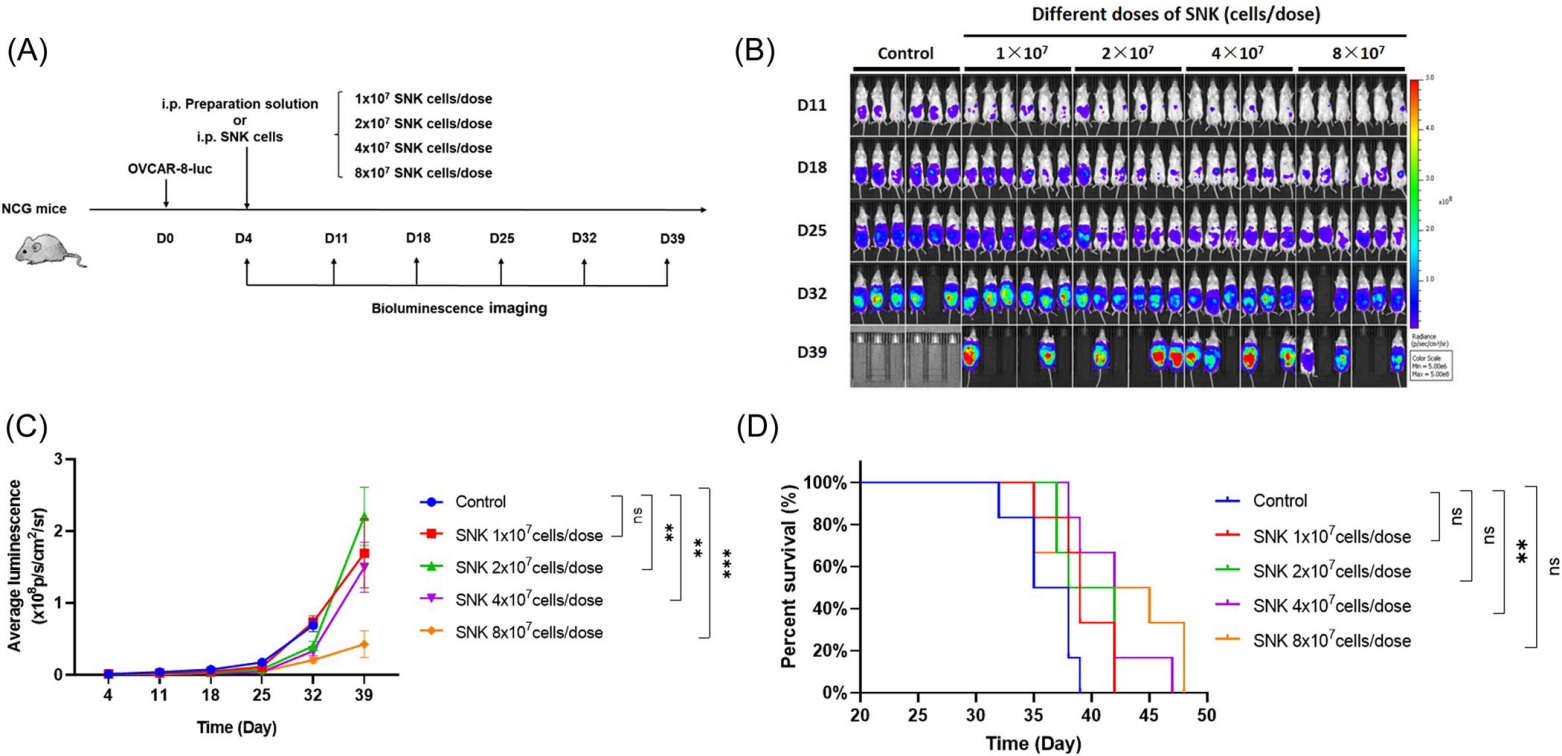
SNK cells demonstrate dose‐dependent antitumor activity in NCG mice. (A) Experimental design for intraperitoneal adoptive transfer of SNK cells into xenograft NCG mice. (B) Bioluminescence imaging was used to measure tumor burden after different doses of SNK cells were transferred for 39 days. (C) Summary of bioluminescence measurements for each group at the indicated times. (D) Mice were monitored for the Kaplan–Meier survival curve, and significance was determined through the log‐rank test (*n* = 6 mice per group). Data are represented as mean ± SEM. *p < .05, **p < .01, ***p < .001. ns, no significance; SNK, super natural killer.

To investigate the in vivo antitumor activity of SNK cells, we evaluated the cytotoxicity of OVCAR‐8 cells in an immunodeficient mouse xenograft tumor model. Mice were administered tumor cells through intraperitoneal injection. After 4 days, they received an i.p. injection of different doses of SNK cells (Figure 5A) to explore the optimal quantity of SNK cells by evaluating the tumor burden and survival of mice. The intraperitoneal tumor burden was monitored using bioluminescent imaging (Figure 5B). The results showed that the reduction in tumor burden and mouse survival was dose‐dependent on the given SNK cells (Figure 5C,D). The higher the dose of injected SNK cells, the greater the tumor inhibition and the longer the survival.

**Figure 6:**
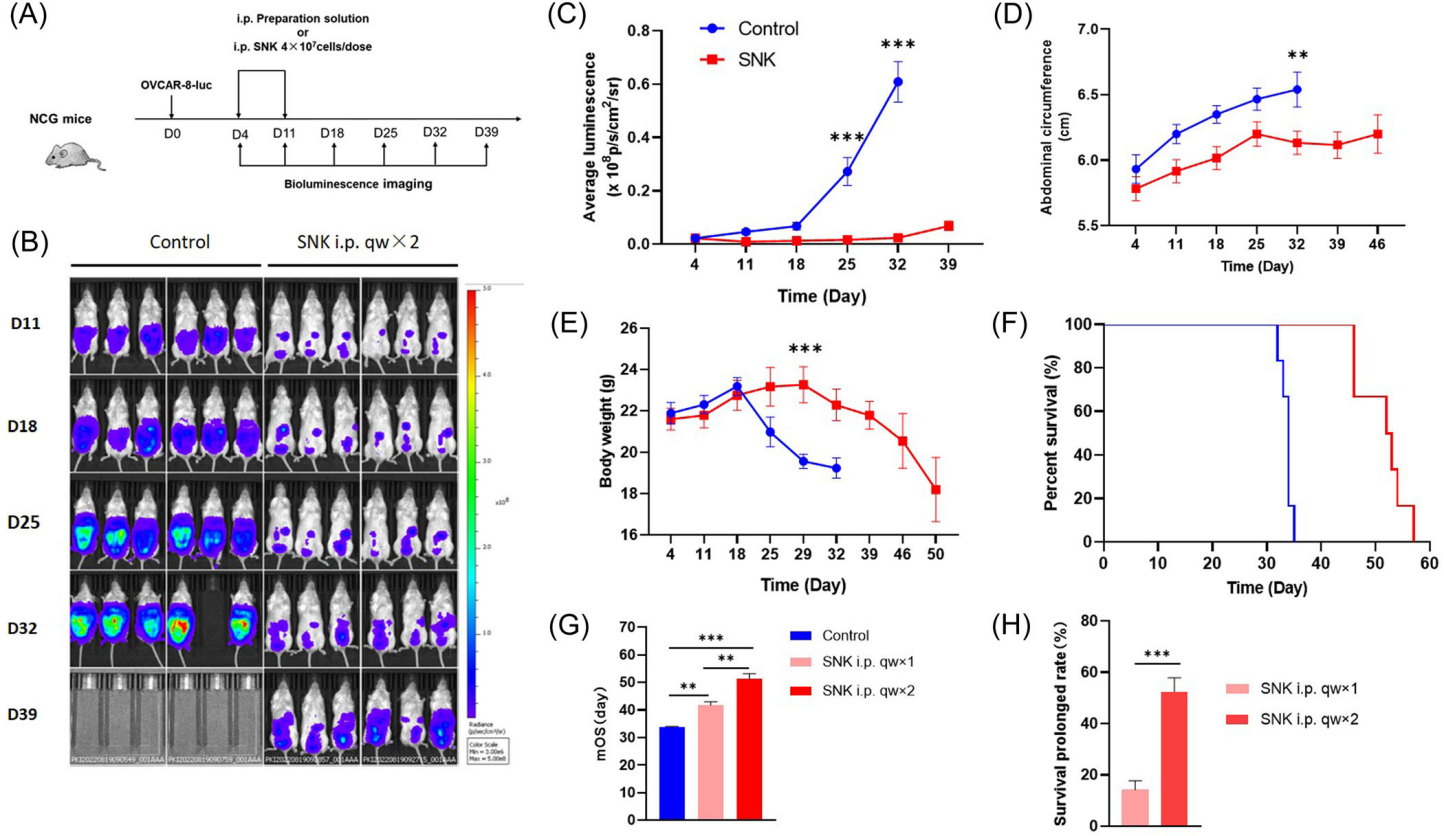
SNK cells demonstrate better antitumor activity and improved survival in vivo. (A) Experimental design for intraperitoneal adoptive transfer of SNK cells into xenograft NCG mice. (B) Bioluminescence imaging was used to determine tumor burden after different doses of SNK cells were transferred for 39 days. (C, D) Summary of bioluminescence measurements for each group at the indicated times (*n* = 6 mice per group). (E) Abdominal circumference was measured for each group at the indicated times. Significant differences were analyzed using one‐way ANOVA. (F, G) Mice were monitored for the Kaplan–Meier survival curve, and significance was determined through the log‐rank test. *n* = 6 mice per group. Data are represented as mean ± SEM. *p < .05, **p < .01, ***p < .001, SNK, super natural killer.

Next, we investigated the in vivo antitumor effects of SNK cells using two i.p. injections (Figure 6A). The decrease in the tumor burden was greater for the SNK cell treatment group than for the control treatment group. Mice receiving the preparation solution demonstrated rapid tumor growth, with most of them reaching a humane experimental endpoint 34 days after enrollment. By contrast, the fluorescence particle count, which indicated the presence of tumors, was significantly lower in the SNK group compared to the control group (0.1615 × 10^8^ p/s/cm^2^/sr vs. 0.2724 × 10^8^ p/s/cm^2^/sr, *p* < .0001) (Figure 6B,C). The abdominal circumference of mice, which reflects ascitic fluid volume, showed slower growth in the SNK cell group than in the control group (6.133 cm vs. 6.54 cm, *p* = .0048) (Figure 6D). Moreover, the body weight loss in the mice in the SNK cell group was slower than that in the control group (Figure 6E), and the overall survival of tumor‐bearing mice infused with SNK cells was significantly prolonged (52.5 days vs. 34.0 days, *p* = .0015) (Figure 6F). Furthermore, the median survival time of mice was elevated by using different frequencies of SNK cells (Figure 6G), and the rate of prolonged survival in mice that received SNK cells twice was significantly higher (52.46 ± 5.42% vs. 14.17 ± 3.51%) than that of the group that received the SNK cells once (Figure 6H).

To elucidate the duration of SNK survival in various tissues of mice subsequent to *i.p* injection, flow cytometric analysis was performed on samples obtained at 0 h, 2 h, 4 h, 24 h, 48 h, 72 h, 7 days, and 14 days following i.p. administration of SNK cells to mice. It was discovered that after i.p. administration of SNK cells into mice, the SNK cells demonstrated a significant accumulation in the abdominal cavity, followed by a gradual dispersion to other tissues. Furthermore, it was shown that approximately 40% of NK cells remained detectable in the abdominal cavity on Day 14 (Figure S3A). In contrast, minimal cellular distribution was observed in other tissues, including blood, liver, spleen, ovary, kidney, and duodenum, with only small quantities of detectable NK cells (Figure S3B–G). By Day 14, the presence of NK cells was scarcely discernible in all tissues.

### 3.6 SNK cells showed increased cytokine signal activation

The aforementioned experiments validated the significant superiority of SNK over PBNK in tumor‐killing function, cytokine secretion capability, and activation/inhibition marker expression. To further investigate the potential regulatory mechanism underlying the disparity in antitumor activity between SNK and PBNK, we conducted RNA sequencing on purified PBNK (resting NK) and SNK (activating NK) cells from three healthy individuals.

Differential gene analysis identified 8886 differentially expressed genes, with log2|FC| > 1 and a *Q* value < 0.05; among these genes, 3854 were upregulated, and 5032 were downregulated (Figure 7A). The GO, KEGG, and REACTOM analyses indicated that the upregulated genes were enriched in the cell cycle and in DNA replication and reparation (Figure 7B–D). The results of gene set enrichment analysis (GSEA) revealed that nucleotide metabolism, fatty acid, and ATP metabolism were significantly enriched in SNK cells (Figure 7E,F). Additionally, SNK significantly elevated the expression of genes involved in the cytokine and chemokine signaling pathways (e.g., *CCL3*, *CCL4*, *CCL23*, *CCR2*, *CCR5*, *CCR7*, and *CXCR6*) (Figure 7G), immune molecules (e.g., *CD96*, *CD226*, *LAG3*, and *TIGIT*) (Figure 7H), cytotoxicity‐related genes (e.g., *GZMA*, *GZMB*, *TGFBR1*, *TGFBR2*, and *TGFBR3L*) and signaling by ILs (e.g., *IL2RG*, *IL21R*, *IL‐22*, *CD80*, and *CISH*) (Figure 7I). Finally, based on the heatmap of significantly differentially expressed genes in fatty acid metabolism, it was found that SNK demonstrated a significantly elevated expression of genes about fatty acid metabolism (e.g., *SLC25A17*, *ALOX5AP*, *ACAT2*, and *MSMO1*) (Figure 7J).

Figure 7Differentially expressed genes between PBNK and SNK cells were analyzed through RNA sequencing. (A) Volcano plot of differential genes between SNK and PBNK cells. The upregulated genes were analyzed using (B) GO, (C) KEGG, and (D) REACTOM enrichment analyses. (E–H) GSEA demonstrated the glucose, fatty, nucleoside, and ATP metabolic pathways of different genes. Heatmap showing differentially expressed genes in the (G) cytokine and chemokine signaling pathway, (H) immune molecules, (I) signaling by interleukins, and (J) fatty acid metabolism. PBNK, natural killer in peripheral blood; SNK, super natural killer.

**Figure:**
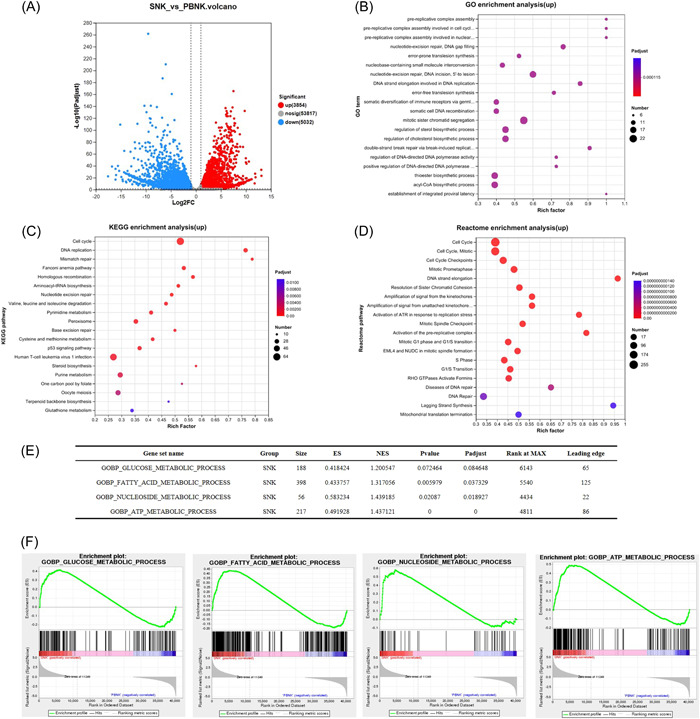

**Figure:**
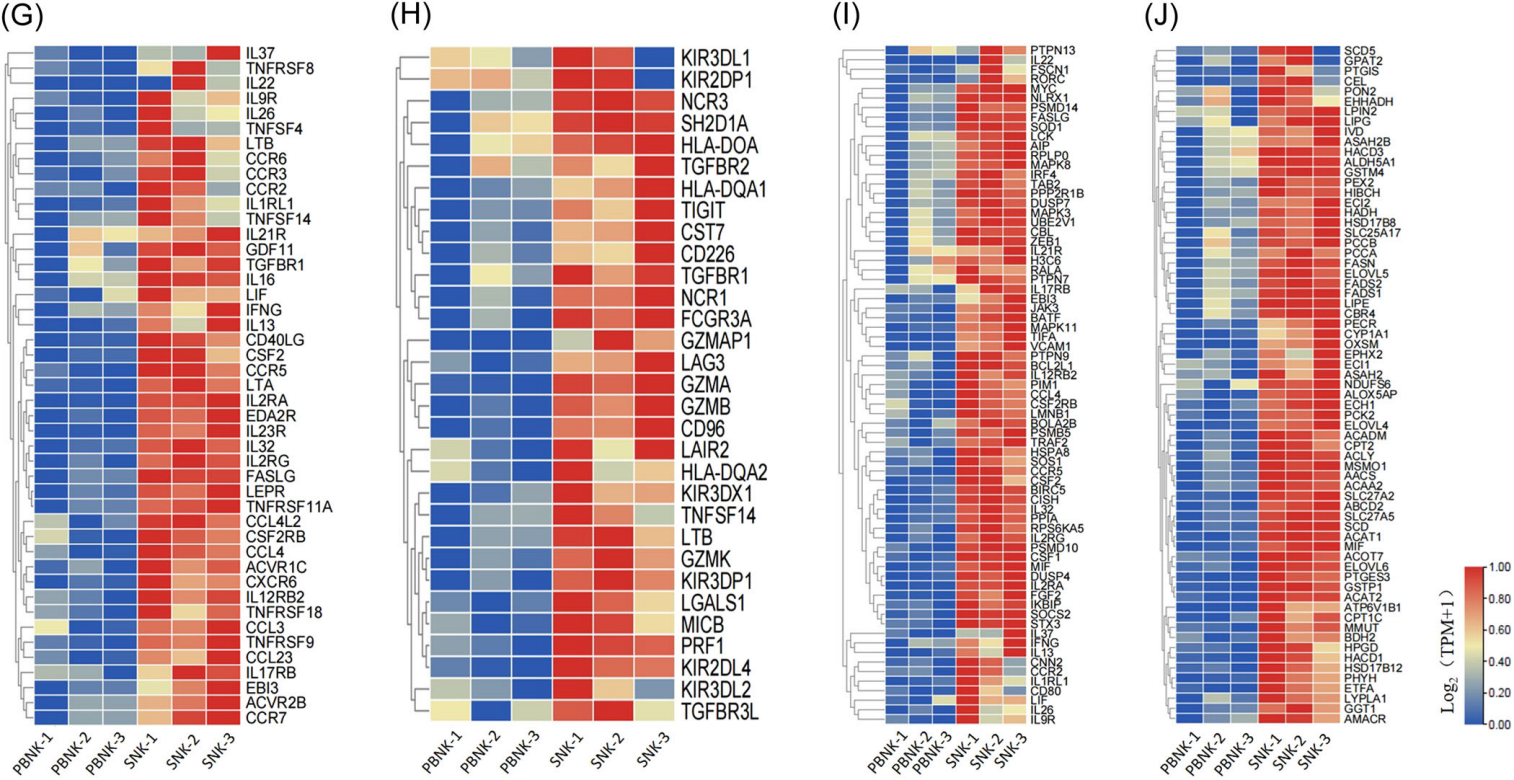

## 4 DISCUSSION

NK cells are lymphocytes known for their capacity to kill malignant cells while avoiding healthy cells. These cells have been expanded and activated ex vivo using irradiated HFWT Wilms' tumor cells,^33^ gene‐modified K562 leukemia cells,^34^ B‐lymphoblastoid cell lines,^35^ or autologous feeder cells.^36^ However, NK cell expansion using feeder cells is complicated and subject to viral or bacterial infections. Thus, in this study, we developed NK cells using a pure cytokine activation and expansion system with a DCTY NK serum‐free medium culture kit, and these cells demonstrated higher expansion efficiency and purity than pure cytokine NK cell expansion systems in the literature.^26^

The cytotoxic activity of NK cells is determined by the balance between inhibitory and activating receptors, which do not require tumor‐specific antigens or antigen‐presenting cell activation.^37^ The present study examined NK cell‐activating receptors, including the C‐type lectin superfamily (CD69, NKG2D, and NKG2C), natural cytotoxicity receptors (NKp30, NKp44, NKp46, and NKp80), DNAX accessory molecule‐1 (DNAM‐1), CD57, and CD94 (Figure 2). We found that activating receptors, such as CD69, NKG2D, and NKP44, were significantly upregulated, which was the same as that in expanded NK cells derived from PBMCs using a feeder cell‐free expansion system.^26^ In the present study, the expression of the activating receptor NKG2C was also higher than that of PBNK cells. This finding differs from that of Chen et al.,^26^ with no change between expanded NK and PBNK cells but consistent with that of Yang et al.,^35^ who expanded primary NK cells using a feeder cell‐free expansion system. Notably, the expression of the inhibitory receptor CD96 was hyper expressed compared with that in PBNK cells, which was similar to that reported by Chen et al.,^26^ demonstrating that the activity of NK cells was derived from the balance of activating and inhibitory receptors.

Notably, activated NK cells lyse adjacent tumor cells mainly by releasing abundant cytotoxic components containing granzymes, perforin, and granlysin.^38^ Granzymes can enter target cells via perforin pores in the plasma membrane or through endocytosis and perforin‐aided escape from endosomes. Granzymes can then induce mitochondrial dysfunction, caspase activation, or caspase‐independent apoptosis for target cells.^38^, ^39^ Furthermore, the antitumor cytotoxicity of NK cells can be mediated by the surface expression of FasL or TRAIL, which can engage and activate their respective receptors on target cells.^39^ Another crucial function of NK cells is the production of cytokines such as IFN‐γ and TNF‐α, which can promote innate and adaptive immunity by recruiting and activating other cells of the immune response, such as monocytes or macrophages, dendritic cells, T cells, and B cells.^40^ Notably, we observed a significant increase in the expression of granzymes, perforin, granlysin, sFasL, IFN‐γ, and TNF‐α in the SNK cells (Figure 3). This significant increase is possibly the main reason for an enhancement in the antitumor potency of SNK cells after expansion (Figure 4).

Adoptively transferred NK cells have shown limited antitumor activity in tumor‐bearing mouse models,^26^, ^35^ in which mice were administered a high dose of rhIL‐2 (50,000 IU/mouse) every other day to facilitate prolonged retention of human NK cells. In our study, mice were not administered IL‐2 because only SNK cells demonstrated better antitumor activity and improved mouse survival (Figures 5 and 6).

Based on the findings of our experiments, a pronounced disparity in lethality between SNK and PBNK cells was observed. To understand the fundamental molecular mechanisms, we examined the regulation of gene expression using RNA‐seq analysis. The analysis revealed that the differentially expressed genes were predominantly enriched in cellular processes, signal transduction, immune system, and cellular metabolism (Figure 7).

NK cells activate specific metabolic pathways in response to the increased energetic demands and biosynthetic demands that occur during immune reactions.^41^ The GSEA of SNK and PBNK suggested that cell metabolic activity, particularly nucleotide and lipid metabolism, was enriched in SNK cells. Further analysis suggested that cholesterol‐regulating genes (*MSMO1, ACAT2*, and *AACS*), fatty acid transport genes (*SLC25A17* and *SLC27A2*), and arachidonic acid metabolism gene *ALOX5AP* were significantly higher in SNK than in PBNK. NK cells engage in the activation of several metabolic pathways to meet the heightened energy and biosynthetic requirements associated with immune responses. An effective strategy for improving NK cell treatment involves further studying the key regulatory molecules involved in NK cell metabolism and using them as targets for genetic alteration, enhancing their ability to destroy tumors. While several trials have validated the efficacy of NK cell treatment, yet there is always potential for future enhancement.

Another potential approach to advance NK cell treatment is integrating combination therapy measures against cancer cells. Recently, studies demonstrated that the combined administration of expanded NK cells and PD1‐blockade demonstrated a significant synergistic effect in effectively eradicating lung tumors in patients.^42^, ^43^ Hasan et al. report that there is a negative correlation between the level of TIGIT and both the release of IFN‐γ and the degranulation of NK cells. This suggests that the utilization of a TIGIT antibody in conjunction with high levels of NK cells may enhance therapeutic efficacy.^44^ Moreover, it is essential to investigate the potential for combining NK cell therapy with traditional cancer treatment strategies like radiation and chemotherapy to enhance the effectiveness of tumor treatment in future studies. According to research done by Jung et al.,^45^ the inclusion of radiotherapy and cisplatin therapy as concurrent chemoradiotherapy regimens has shown a notable improvement in the ability of NK cells to kill cancer cells. Furthermore, Thangaraj et al.^46^ have uncovered that using the daratumumab, bortezomib, and dexamethasone regimen can enhance the antitumor effectiveness of NK cells in a xenograft mice model of multiple myeloma.

Our study offers novel insights into the mechanism of SNK cells derived from PBMCs, which demonstrate strong antitumor activity, and provide directions for further research. However, our study has limitations. First, although we primarily analyzed markers of the NK cell surface, we did not investigate all the activating and inhibitory markers of NK cells. Thus, further research should be conducted to examine these specific markers. Moreover, we found that SNK cells without IL‐2 showed antitumor activity in vitro and in vivo, but previous studies have found that NK cells with IL‐2 exhibited antitumor activity. Thus, future studies should investigate the cytokine (e.g., IL‐2 or IL‐15) to enhance the antitumor effects of NK cells in vivo. Consequently, based on the focus of this study, we plan to conduct further research and analyses of glucose and fatty acid metabolic processes to identify pivotal targets associated with NK cell function.

## 5 CONCLUSION

In summary, PBMC‐derived SNK cells were sufficiently activated and exhibited significantly enhanced antitumor activity. Moreover, because NK cell‐based transfer strategies have been shown to be safe without causing toxicity in multiple cancer types, the adoptive transfer of NK cells is a promising immunotherapeutic approach for patients with ovarian cancer.

## AUTHOR CONTRIBUTIONS

**Chuanling Liu**: Data curation; formal analysis; investigation; methodology; software; validation; visualization; writing—original draft. **Yingying Li**: Data curation; formal analysis; investigation; methodology; software; validation; visualization. **Yanrong Li**: Data curation; formal analysis; investigation; methodology; software; visualization. **Meng Hu**: Data curation; investigation; methodology. **Haiyan Wang**: Data curation; investigation; methodology. **Shasha Lu**: Data curation; investigation; methodology. **Zhao Li**: Data curation; investigation; methodology. **Dilinuer Dilimulati**: Data curation; investigation; methodology. **Shunchang Jiao**: Funding acquisition; project administration; resources; supervision. **Shelian Lu**: Conceptualization; investigation; project administration; resources; supervision; validation; writing—original draft; writing—review and editing. **Weihong Zhao**: Project administration; resources; supervision.

## CONFLICT OF INTEREST STATEMENT

The authors declare no conflict of interest.

## ETHICS STATEMENT

The animal study protocol was approved by the Yicon(Beijing) Biomedical Technology Lnc.IACUC[YK‐IACUC‐2022‐0012].

## Supporting information

Supplementary information.

Supplementary information.

Supplementary information.

Supplementary information.

## Data Availability

All data generated or analyzed during this study are included in this published article.
