# Supplementary figures and images for: Sufficiently activated mature natural killer cells derived from peripheral blood mononuclear cells substantially enhance antitumor activity
Source: Immun Inflamm Dis. 2024 Jan 10;12(1):e1143. doi: 10.1002/iid3.1143 (PMC10777885; doi:10.1002/iid3.1143)

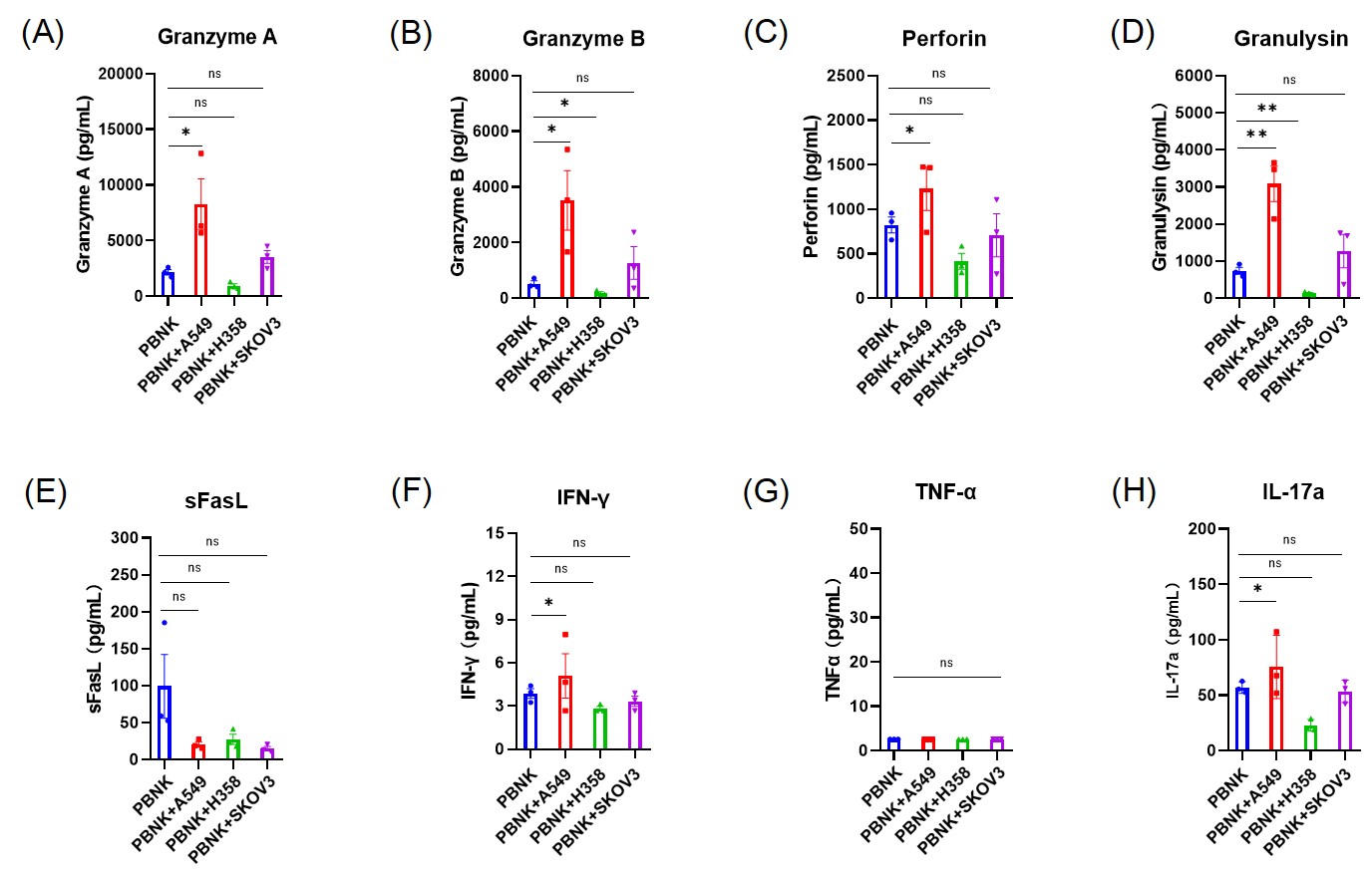

Supplement: Supplementary file 1 — Supplementary information. [file IID3-12-e1143-s001.jpg]

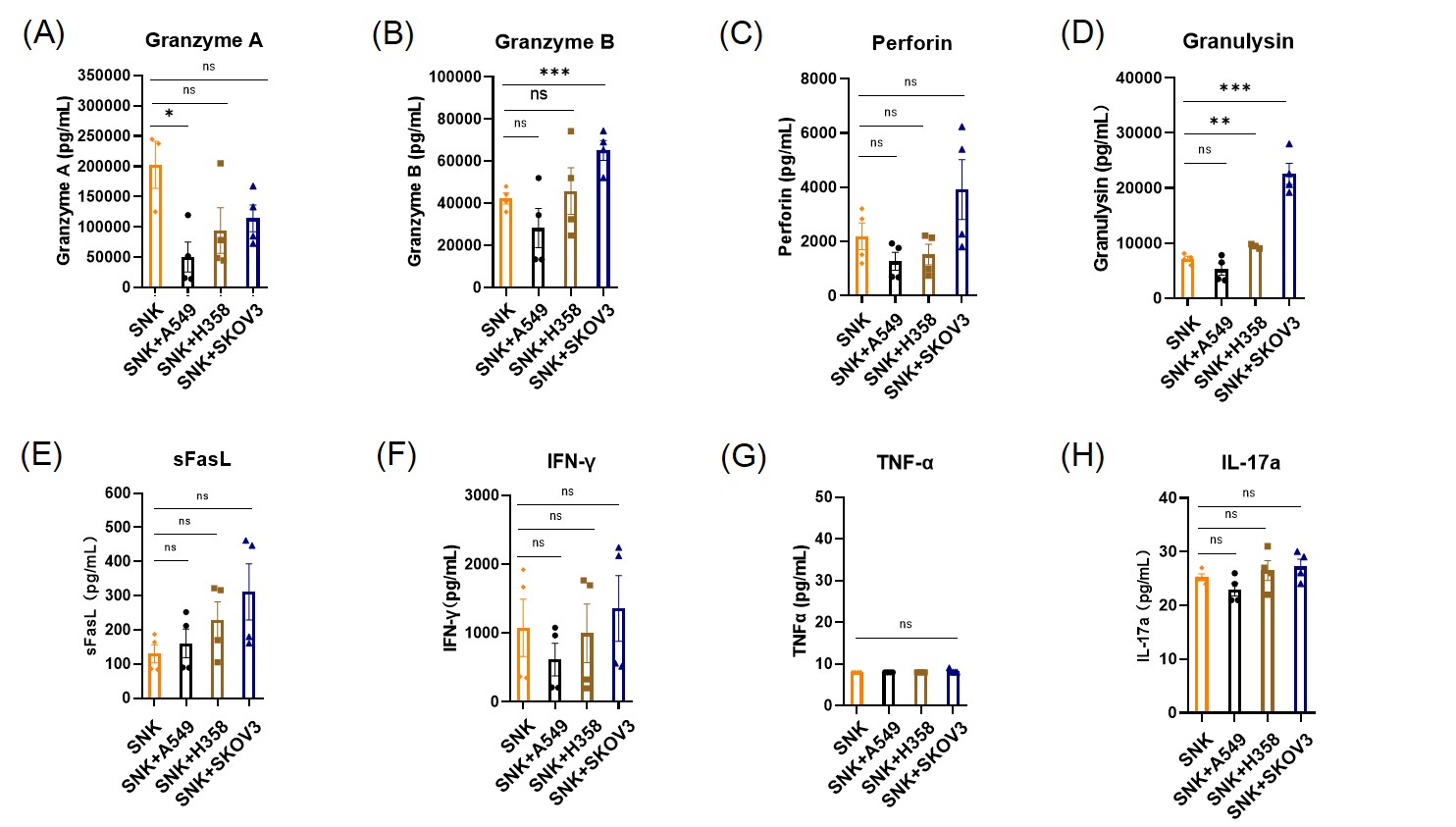

Supplement: Supplementary file 2 — Supplementary information. [file IID3-12-e1143-s004.jpg]

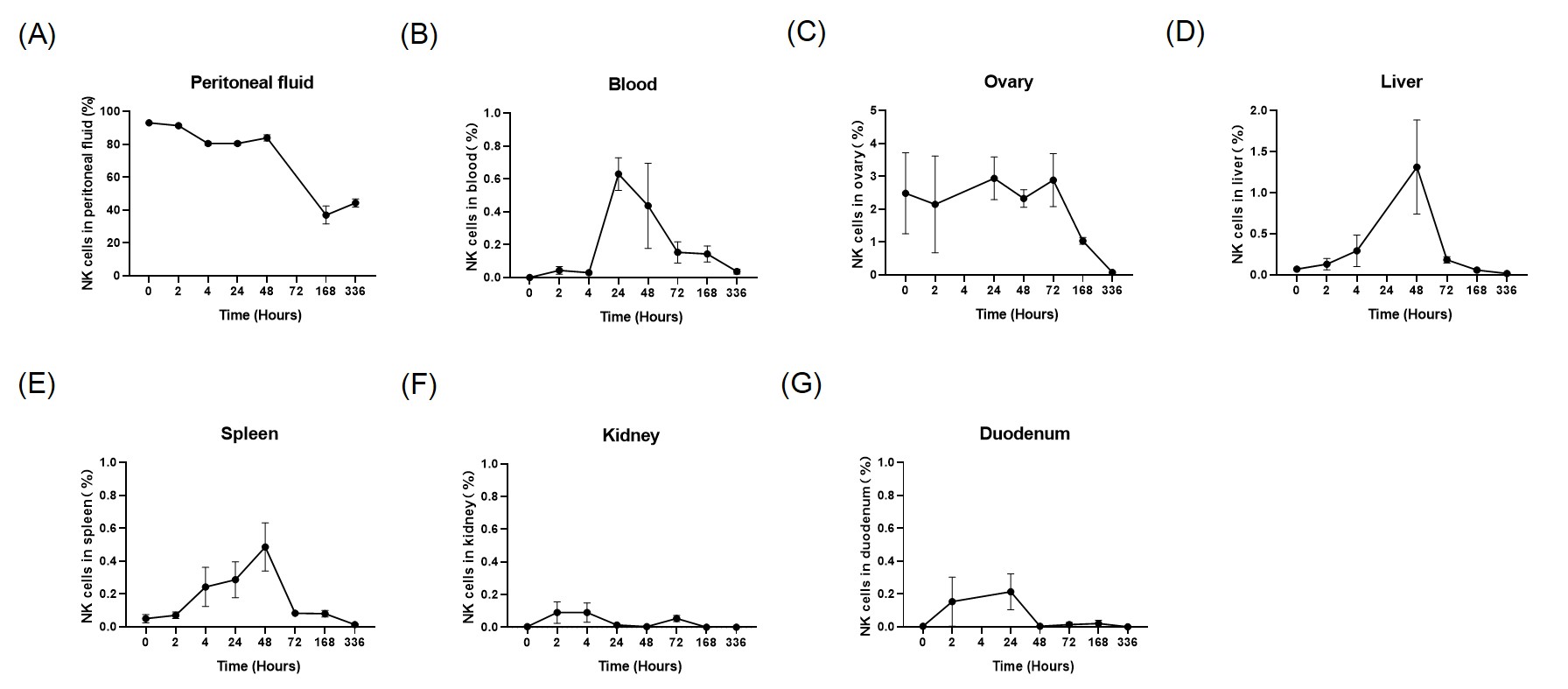

Supplement: Supplementary file 3 — Supplementary information. [file IID3-12-e1143-s003.jpg]
