# Supplementary material for: Sufficiently activated mature natural killer cells derived from peripheral blood mononuclear cells substantially enhance antitumor activity
Source: Immun Inflamm Dis. 2024 Jan 10;12(1):e1143. doi: 10.1002/iid3.1143 (PMC10777885; doi:10.1002/iid3.1143)
Supplement: Supplementary file 4 — Supplementary information. [file IID3-12-e1143-s002.docx]

Supplementary Figures:

Supplemental Figure 1. PBNK cells soluble secretion in the presentce of target cancer. To investigate the secretion of PBNK soluble factors in the presence of target cancer cells, the LEGENDplexTM Human Inflammation Panel (13-plex) was utilized to evaluate the cytokine levels in Resting PBNK cells when co-cultured with H358, A549, and SKOV-3 cells, respectively. The levels of (A) granzyme A, (B) granzyme B, (C) perforin, (D) granulysin, (E) sFasL, (F) IFN-γ, (G) TNF-α, were evaluated in the supernatants of various cells that were stimulated with cytokine-free medium for a duration of 24 hours. Statistical analysis was performed using one-way ANOVA. Data are shown as mean ± SEM (n = 3). **p* < 0.05, ***p* < 0.01, ****p* < 0.001, ns, no significance.

Supplemental Figure 2. SNK cells soluble secretion in the presence of target cancer. In order to examine the release of soluble factors from SNKs in the presence of cancer cells, the LEGENDplexTM Human Inflammation Panel (13-plex) was employed to assess the cytokine concentrations in SNK cells during co-cultivation with H358, A549, and SKOV-3 cells, respectively.The levels of (A) granzyme A, (B) granzyme B, (C) perforin, (D) granulysin, (E) sFasL, (F) IFN-γ, (G) TNF-α, were assessed in the supernatants of different cells that were stimulated with cytokine-free medium for a 24-hour period. Statistical analysis was performed using one-way ANOVA. Data are shown as mean ± SEM (n = 4). **p* < 0.05, ***p* < 0.01, ****p* < 0.001, ns, no significance.

Supplemental Figure 3. Survival of NK cells in different tissues after intraperitoneal injection. The tissues, were collected at different time points (0h, 2h, 4h, D1, D2, D3, D7, and D14) after SNKs（D20）intraperitoneal administration，and the percents of NK cells in various tissues was assessed using flow cytometry. The time distribution curve of SNK cells in (A) peritoneal fluid, (B) blood, (C) ovary, (D) liver, (E) Spleen, (f) kidney, and (G) duodenum. Statistical analysis was performed using one-way ANOVA. Data are shown as mean ± SEM (n = 3). **p* < 0.05, ***p* < 0.01, ****p* < 0.001, ns, no significance.

Supplementary Method

In vivo biodistributions

The SNK cells were cultured until reaching D20, and subsequently, the cell density was adjusted to 1×10^8^ cells/mL using a compound electrolyte injection. Each NPG mouse received an intraperitoneal injection of 4×10^7^ cells/400μL per mouse. The tissues, including peritoneal fluid, orbital blood, spleen, liver, kidney, ovary, and duodenum, were gathered at various time intervals (0h, 2h, 4h, D1, D2, D3, D7, and D14) subsequent to intraperitoneal administration. Next, the peritoneal fluid underwent centrifugation to eliminate cellular components. The erythrocytes were subsequently lysed using OptiLyse C Lysing Solution (Beckman Coulter, Brea, CA, USA). The remaining tissues were subjected to two washes with PBS and subsequently sectioned and digested with collagenase II (Gibco, Waltham, MA, USA). for a duration of 15-20 minutes. Subsequently, all cells were subjected to staining using specific antibodies (BV605-CD45, FITC-CD3, and PE-CD56, all from BioLegend). Following incubation in darkness at room temperature for 15 minutes, the stained cells were washed with PBS. The stained and fixed cells were analyzed using a BD LSRII flow cytometer (BD Biosciences, San Jose, CA, USA) and FlowJo software (Tree Star, Ashland, OR, USA). CD56^+^CD3^−^ populations were evaluated in the NK cells.
